# The Effect of Anabolic Steroid Administration on Passive Stretching-Induced Expression of Mechano-Growth Factor in Skeletal Muscle

**DOI:** 10.1155/2013/313605

**Published:** 2013-08-25

**Authors:** Satoshi Ikeda, Yurie Kamikawa, Akihiko Ohwatashi, Katsuhiro Harada, Akira Yoshida

**Affiliations:** ^1^Department of Rehabilitation and Physical Medicine, Graduate School of Medical and Dental Sciences, Kagoshima University, 8-35-1 Sakuragaoka, Kagoshima 890-8506, Japan; ^2^School of Medical Sciences, Faculty of Medicine, Kagoshima University, 8-35-1 Sakuragaoka, Kagoshima 890-8544, Japan

## Abstract

*Background*. Stretching of skeletal muscle induces expression of the genes which encode myogenic transcription factors or muscle contractile proteins and results in muscle growth. Anabolic steroids are reported to strengthen muscles. We have previously studied the effects of muscle stretching on gene expression. Here, we studied the effect of a combination of passive stretching and the administration of an anabolic steroid on mRNA expression of a muscle growth factor, insulin-like growth factor-I autocrine variant, or mechano-growth factor (MGF). *Methods*. Twelve 8-week-old male Wistar rats were used. Metenolone was administered and passive repetitive dorsiflexion and plantar flexion of the ankle joint performed under deep anesthesia. After 24 h, the gastrocnemius muscles were removed and the mRNA expression of insulin-like growth factor-I autocrine variant was measured using quantitative real-time polymerase chain reaction. *Results*. Repetitive stretching in combination with metenolone, but not stretching alone, significantly increased MGF mRNA expression. *Conclusion*. Anabolic steroids enhance the effect of passive stretching on MGF expression in skeletal muscle.

## 1. Introduction

The musculoskeletal system is a fundamental structure required for movement and daily living. Muscle strengthening is very important in rehabilitation medicine, sports medicine, geriatric medicine, and other fields of medicine. Various methods, including weight bearing, endurance training, and manual resistance training, are used to strengthen the muscles. Muscle strengthening training is also used in the clinical rehabilitation of various diseases. However, muscle strengthening is difficult to achieve in patients who are unconscious or suffering from paralysis.

Various factors are reported to enhance the growth of skeletal muscle tissue. The humoral factors that have been identified as muscle growth factors are fibroblast growth factor (FGF), the insulin-like growth factors (IGF-I and -II), and transforming growth factor-beta (TGF-*β*) [1]. Activation of the PI3 kinase pathway can induce skeletal muscle hypertrophy, defined as an increase in skeletal muscle mass. In mammals, skeletal muscle hypertrophy results from an increase in the sizes of preexisting skeletal muscle fibers rather than in the number of fibers. The effects of the PI3 kinase pathway on skeletal muscle have been detected most prominently downstream of IGF-I signaling [2]. Recently, mechanical stimuli have been implicated in the regulation of skeletal muscle mass and the maintenance of muscle mass [3, 4]. 

The mechanism of mechanotransduction has been elucidated. Skeletal muscle stretch/overload increases the mRNA expression of IGF-I, particularly the specific autocrine IGF-I splicing variant mechano-growth factor (MGF) [4]. Mechanical environment and changes in muscle structure and physiology suggest that there may be pathways within muscle cells through which mechanical signals can be converted into chemical signals that in turn generate numerous, specific downstream events that determine the muscle's form and function [5]. Several studies have reported that muscle stretching induces muscle growth and hypertrophy [6–8]. Such mechanical stimulation is thought to be helpful for patients with muscle weakness and also applicable to clinically unconscious patients and those with paralysis.

Administration of anabolic steroids also produces muscle hypertrophy. Anabolic steroids have been shown to increase lean body mass, induce muscle fiber hypertrophy in a dose-dependent manner, and increase muscle strength [9]. Recently, anabolic steroids have been administered to treat muscle weakness in patients with various chronic diseases [10, 11]. Therefore, anabolic steroids are thought to have an important role in the treatment of patients with muscle weakness, such as bedridden patients and those with stroke, neuropathy, and many other diseases. However, the effects of anabolic steroids on the results of mechanical stimulation to promote muscle strengthening are not well known. 

Therefore, we studied the effects of the combination of mechanical stimulation and administration of an anabolic steroid on the expression of myogenic transcription factors and muscle growth factor.

## 2. Materials and Methods

### 2.1. Animals and Experimental Groups

We used 8-week-old male Wistar rats (*n* = 12) in this study. The study protocol was carried out in accordance with the Guide for Animal Experimentation of the faculty in the Department of Medicine of Kagoshima University and the guidelines of the United States National Institute of Health, and this study was approved by the animal experiment committee of Kagoshima University. The animals were housed in plastic cages in an environmentally controlled room with a 12/12-hour light-dark cycle, and food and water were provided ad libitum. The rats in the treatment group (*n* = 6) were anesthetized with sodium pentobarbital (40 mg/kg) administered intraperitoneally contractile and the anabolic steroid metenolone (20 mg/kg) was injected intramuscularly. Then, the right gastrocnemius muscles were stretched repeatedly by manual ankle dorsiflexion 15 times per minute for 15 min. The contralateral muscles were not stretched as a control. In the control rats (*n* = 6), the gastrocnemius was stretched as for the treatment group, but no metenolone was injected. Twenty-four hours after the procedure, the rats were sacrificed by injection of a lethal dose of sodium pentobarbital and their medial gastrocnemius muscles removed on both sides. The removed muscles were immediately preserved in liquid nitrogen and stored at −80°C for RNA extraction.

### 2.2. RNA Isolation and Analysis

The tissues were homogenized with Trizol reagent (10 mL/mg tissue; Invitrogen, Carlsbad, CA, USA) using a hand-held homogenizer, and chloroform (0.2x the volume of the tissue) was added. The total RNA remained in the supernatant after extraction and after the removal of all protein and deoxyribonucleic acid by using a precipitator. The total RNA concentration was estimated spectrophotometrically at a wavelength of 260 nm, and the sample was divided into 10 ng aliquots. We confirmed the purity of the RNA and identified the 18S and 28S ribosomal bands by staining with ethidium bromide and viewing under ultraviolet light.

### 2.3. cDNA Synthesis

Aliquots of mRNA (10 ng) were washed twice with 75% ethanol and dissolved in 50 *μ*L of diethylpyrocarbonate- (DEPC-) treated water (0.2 *μ*g cDNA/*μ*L). The cDNA synthesis mixture consisted of 1 ng of mRNA, 2.5 *μ*M (*μ*L) oligo-dT, and 7 *μ*L of DEPC-treated water in a total volume of 13 *μ*L. The mixture was incubated at 65°C for 10 min and immediately cooled on ice. Then, 20 U (40 U/*μ*L) of dNTP mix, MgCl_2_ to 3 mM, 20 U (40 U/*μ*L) of Protector RNase inhibitor, and 10 U of Transcriptor Reverse Transcriptase were added to the incubated mixture to make a total volume of 20 *μ*L. Every round of cDNA synthesis was completed by incubation at 60°C for 30 min.

### 2.4. Oligonucleotide Primers

The sequences used were derived from the following genes: MGF (forward: GCATTGTGGATGAGTGTTGC; reverse: CTTTTCTTGTGTGTCGATAGG); rat MyoD (forward: GGAGACAATCCTCAAGCGATGC; reverse: AGCACCTGGTAAATCGGATTGG); rat myogenin (forward: ACTACCCACCGTCCATTCAC; reverse: TCGGGGCACTCACTGTCTCT); rat glyceraldehyde-3-phosphate dehydrogenase (GAPDH) (forward: TGGTGAAGGTCGGTGTGAAC; reverse: AGGGGTCGTTGATGGCAACA).

### 2.5. Analysis by Quantitative Real-Time Polymerase Chain Reaction (RT-qPCR)

The polymerase chain reaction (PCR) procedure was performed using a Light Cycler (Roche Diagnostics, Indianapolis, IN, USA) thermalcycler with the following protocol: 5 min at 95°C, followed by 45 cycles of 10 s at 94°C, 5 s at 58°C, and 10 s at 72°C. After these amplification steps were completed, the melting temperatures of the products were examined. The specificity of each reaction was measured by observing the presence of a single reaction product on an agarose gel and a single peak on the DNA melting temperature curve generated at the conclusion of the reaction. The quantity of DNA was determined by detection of the fluorescent dye SYBR green at the point of extension. The ratios of the amounts of MGF, MyoD, and myogenin mRNA to that of GAPDH as the internal control were calculated and compared between the legs within each group and between the groups.

### 2.6. Statistical Analysis

All values are expressed as the mean ± standard error (SE). The Wilcoxon signed-rank test was used to compare data between the stretched right muscles and unstretched left muscles of the same animals within each group. The level of significance was set at 5% or less (*P* ≤ 0.05).

## 3. Results

**Figure 1:**
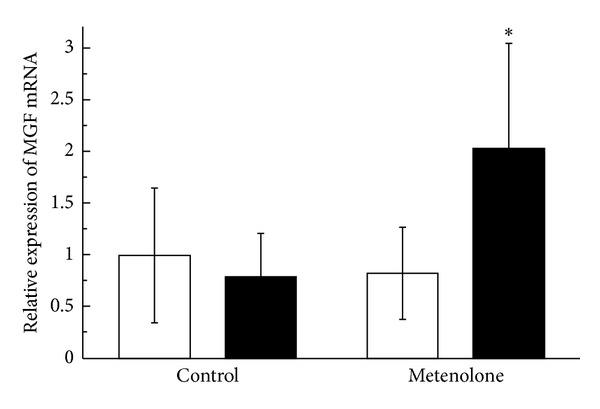
Relative mRNA expression ratio of MGF. The open bars indicate the hind limbs on the unstretched side and the closed bars those on the stretched side (*P < 0.05).

**Figure 2:**
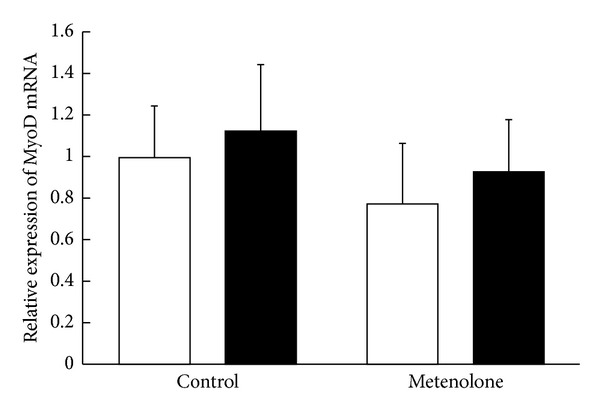
Relative mRNA expression ratio of MyoD. The open bars indicate the hind limbs on the unstretched side and the closed bars those on the stretched side.

**Figure 3:**
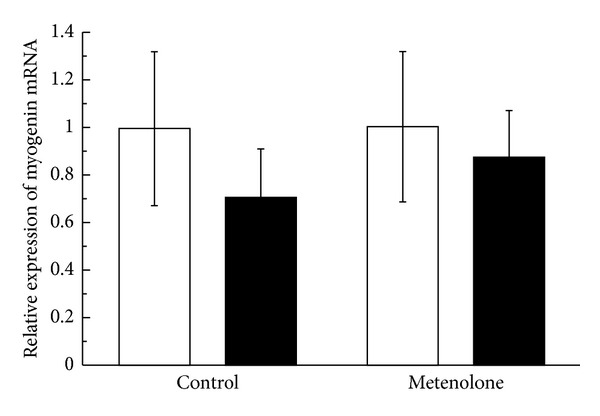
Relative mRNA expression ratio of myogenin. The open bars indicate the hind limbs on the unstretched side and the closed bars those on the stretched side.

The relative mRNA expression ratio of MGF in the muscles from the repetitively stretched side of the metenolone-treated animals was 2.03 ± 1.03 (mean ± SE). This value was significantly higher than that of the unstretched side of the treated animals or of either side of the group not treated with metenolone (*P* = 0.028). The MGF expression ratio in the muscles of the unstretched side of the metenolone-treated rats was 0.83 ± 0.45, which did not differ significantly from both the stretched or the unstretched side of the control group not treated with metenolone. There was no increase in the stretched side relative to the unstretched side in the group not treated with metenolone. The ratio was 0.79 ± 0.42 (Figure 1). The relative mRNA expression ratio of MyoD in the muscles from the repetitively stretched side of the metenolone-treated rats was 0.93 ± 0.25. There was no significant difference in comparison with the unstretched side of the treated rats or with either side of the rats not treated with metenolone. The MyoD expression ratio in muscles from the unstretched sides of the metenolone-treated rats was 1.13 ± 0.32, which did not differ significantly from either the stretched or unstretched side of the control group not treated with metenolone. There was no increase in MyoD expression in the stretched side relative to the unstretched side in the group not treated with metenolone. The ratio was 0.78 ± 0.29 (Figure 2). The relative mRNA expression ratio of myogenin in the muscles from the repetitively stretched side of metenolone-treated rats was 0.88 ± 0.20, which did not differ significantly from the unstretched side of the treated rats or from either side of the group not treated with metenolone. The expression ratio of myogenin in the muscles from the unstretched side of the metenolone-treated rats was 1.01 ± 0.32, which did not differ significantly from either the stretched or the unstretched side of the control group not treated with metenolone. The mRNA expression ratio of myogenin was 0.71 ± 0.20. There was no increase in the stretched side relative to the unstretched side in the group not treated with metenolone (Figure 3).

## 4. Discussion

We studied the effects of anabolic steroid administration on the efficacy of mechanical stimulation of skeletal muscle. A single 15-minute session of repetitive stretching of the gastrocnemius muscle after the administration of the anabolic steroid metenolone significantly increased the mRNA expression of MGF, whereas there was no such increase in the unstretched muscles of anabolic steroid-treated animals or in either the stretched or unstretched muscles of animals not treated with the anabolic steroid. 

Mechanical stimulation has been reported to promote the growth of skeletal muscle. Vandenburgh and Kaufman showed hypertrophic effects in skeletal muscle cells cultured on elastic film under tension [12]. Carson et al. reported that stretching the anterior latissimus dorsi muscles of quail induced hypertrophy of the stretched muscles [13]. 

Skeletal muscle differentiation and growth are controlled by myogenic transcription factors. These factors control muscle development in vertebrates by regulating myoblast proliferation, migration, fusion, and functional adaptation into fast-twitch and slow-twitch fibers. Postnatal hypertrophic growth, remodeling, and functional differentiation are all controlled transcriptionally [14]. Stretching of skeletal muscle induces mRNA expression of the myogenic transcription factors myogenin, MyoD, and MRF4 [15]. 

Mechanical stimuli play a major role in the regulation of skeletal muscle mass, and the maintenance of muscle mass contributes significantly to the prevention of disease and issues associated with quality of life. The mechanisms involved in converting mechanical signals into molecular events. Recent studies have revealed that signaling through a protein kinase called the mammalian target of rapamycin (mTOR) plays a central role in this process [3].

McKoy et al. reported that stretching of skeletal muscle induced the expression of MGF, an autocrine variant of insulin-like growth factor-1 with an important role in local growth or hypertrophy in response to mechanical stimulation of skeletal muscle [16]. The effect of anabolic steroids on the expression of MGF in response to stretching of skeletal muscle was not well known. We found that administration of an anabolic steroid worked synergistically with repetitive stretching to increase the mRNA expression of MGF. This finding suggests that anabolic steroids enhance the effect of stretching on muscle growth by increasing the production of MGF.

The duration of stretching has been reported to influence its effect on muscle growth. Stretching for 1 hour induced mRNA expression of myogenin [7]. The level of MyoD mRNA had increased relative to that of the control group 24 hours after a single session of stretching for 30 minutes, whereas the expression level of atrogin-1 increased after 2, 3, and 7 stretches [17]. The expression of MyoD in the soleus had increased for 24 hours after stretching for 15 minutes [18]. Either continuous or repetitive short-duration stretching of muscles for 1 week increased the mRNA expression levels of MyoD, myogenin, and embryonic MyHC relative to those of unstretched muscles [6]. In clinical rehabilitation, the duration of treatment tends to be short. In this respect, the 15 minutes of passive stretching performed in our study resembles the stimulus applied during clinical rehabilitation. Muscle maintenance is important in patients with various diseases. Muscle maintenance by passive stretching is thought to be useful not only in conscious patients with a variety of conditions but also in unconscious patients. Further studies are planned to explore the potential benefits of combination therapy including anabolic steroids and passive stretching in a clinical rehabilitation setting. 

## 5. Conclusion

Mechanical stimulation of skeletal muscle in conjunction with the administration of an anabolic steroid induced mRNA expression of MGF. This finding suggests that the combination of anabolic steroids with muscle stretching exercise could promote muscle strengthening in patients with muscle weakness.

## References

[1] Allen RE, Boxhorn LK (1989). Regulation of skeletal muscle satellite cell proliferation and differentiation by transforming growth factor-beta, insulin-like growth factor I, and fibroblast growth factor. *Journal of Cellular Physiology*.

[2] Glass DJ (2010). PI3 kinase regulation of skeletal muscle hypertrophy and atrophy. *Current Topics in Microbiology and Immunology*.

[3] Hornberger TA (2011). Mechanotransduction and the regulation of mTORC1 signaling in skeletal muscle. *International Journal of Biochemistry and Cell Biology*.

[4] Zablocka B, Goldspink PH, Goldspink G, Górecki DC (2012). Mechano-growth factor: an important cog or a loose screw in the repair machinery?. *Frontiers in Endocrinology*.

[5] Tidball JG (2005). Invited review: mechanical signal transduction in skeletal muscle growth and adaptation. *Journal of Applied Physiology*.

[6] Kamikawa Y, Ikeda S, Harada K, Ohwatashi A, Yoshida A (2013). Passive repetitive stretching for a short duration within a week increases myogenic regulatory factors and myosin heavy chain mRNA in rats’ skeletal muscles. *The Scientific World Journal*.

[7] Ikeda S, Yoshida A, Matayoshi S, Horinouchi K, Tanaka N (2004). Induction of myogenin messenger ribonucleic acid in rat skeletal muscle after 1 hour of passive repetitive stretching. *Archives of Physical Medicine and Rehabilitation*.

[8] Ikeda S, Yoshida A, Matayoshi S, Tanaka N (2003). Repetitive stretch induces c-fos and myogenin mRNA within several hours in skeletal muscle removed from rats. *Archives of Physical Medicine and Rehabilitation*.

[9] Evans NA (2004). Current concepts in anabolic-androgenic steroids. *The American Journal of Sports Medicine*.

[10] Basaria S, Wahlstrom JT, Dobs AS (2001). Clinical review 138: anabolic-androgenic steroid therapy in the treatment of chronic diseases. *Journal of Clinical Endocrinology and Metabolism*.

[11] Supasyndh O, Satirapoj B, Aramwit P (2013). Effect of oral anabolic steroid on muscle strength and muscle growth in hemodialysis patients. *Clinical Journal of the American Society of Nephrology*.

[12] Vandenburgh H, Kaufman S (1979). In vitro model for stretch-induced hypertrophy of skeletal muscle. *Science*.

[13] Carson JA, Alway SE, Yamaguchi M (1995). Time course of hypertrophic adaptations of the anterior latissimus dorsi muscle to stretch overload in aged Japanese quail. *Journals of Gerontology A*.

[14] Braun T, Gautel M (2011). Transcriptional mechanisms regulating skeletal muscle differentiation, growth and homeostasis. *Nature Reviews Molecular Cell Biology*.

[15] Lowe DA, Alway SE (1999). Stretch-induced myogenin, MyoD, and MRF4 expression and acute hypertrophy in quail slow-tonic muscle are not dependent upon satellite cell proliferation. *Cell and Tissue Research*.

[16] McKoy G, Ashley W, Mander J (1999). Expression of insulin growth factor-1 splice variants and structural genes in rabbit skeletal muscle induced by stretch and stimulation. *Journal of Physiology*.

[17] Gomes AR, Soares AG, Peviani S, Nascimento RB, Moriscot AS, Salvini TF (2006). The effect of 30 minutes of passive stretch of the rat soleus muscle on the myogenic differentiation, myostatin, and atrogin-1 gene expressions. *Archives of Physical Medicine and Rehabilitation*.

[18] Peviani SM, Gomes ARS, Moreira RFC, Moriscot AS, Salvini TF (2007). Short bouts of stretching increase myo-D, myostatin and atrogin-1 in rat soleus muscle. *Muscle and Nerve*.

